# Hierarquizar a Anemia Falciforme nas Repercussões Cardíacas Sempre em Alerta ao Espectro Geral das Anemias Hemolíticas

**DOI:** 10.36660/abc.20220831

**Published:** 2022-11-23

**Affiliations:** 1 Universidade de São Paulo Faculdade de Medicina Instituto do Coração São Paul SP Brasil Instituto do Coração da Faculdade de Medicina da Universidade de São Paulo - InCor-FMUSP, São Paulo, SP – Brasil; 2 Beneficência Portuguesa de São Paulo Departamento de Nefrologia São Paulo MG Brasil Departamento de Nefrologia - Beneficência Portuguesa de São Paulo, São Paulo, SP – Brasil

**Keywords:** Doenças Cardiovasculares, Anemia Falciforme, Anemia Hemolítica, Hemonoglobinopatias/complicações, Hipertensão Pulmonar/complicações, Diagnóstico por Imagem/métodos

O enfoque nas repercussões cardíacas das Anemias Hemolíticas tem sido enfatizado sobremaneira por documentos que alcançam toda a classe médica nacional no presente editorial, tendo tido maiores destaques a Anemia Falciforme^1,2^ e a Talassemia.^3,4^

A doença falciforme é a hemoglobinopatia genética mais frequente em todo o mundo.^5^ Graças ao aprimoramento do manejo médico desses pacientes, sua expectativa de vida melhorou nos últimos anos.^6^ No entanto, as complicações cardiopulmonares continuam sendo uma das principais causas de morte em pacientes adultos com doença falciforme.^7^ O conhecimento atual sobre o envolvimento cardíaco na doença falciforme é derivado principalmente de estudos dos genótipos da anemia falciforme.^8,9^ A hipertensão sistólica pulmonar, avaliada pela elevação da velocidade de regurgitação tricúspide (VRT) e a disfunção diastólica do ventrículo esquerdo diagnosticada por ultrassonografia, têm sido associadas ao aumento da mortalidade e são características da anemia falciforme pela homozigose da hemoglobina S.^8-10^ Por outro lado, a doença da hemoglobina SC (HbSC), resultante da heterozigosidade composta para duas mutações diferentes do gene da betaglobina, tem fisiopatologia diferente^11^ e perfil clínico mais atenuado de apresentação clínica.^12^ Os pacientes com HbSC geralmente têm uma taxa de hemólise relativamente baixa e apenas anemia leve.^11^ Além disso, a prevalência de obesidade é maior em pacientes com HbSC do que na anemia falciforme.^13^ Essa comorbidade não hematológica pode contribuir para a remodelação cardíaca.^14^ Atualmente, a ecocardiografia é recomendada para acompanhamento de rotina de todos os pacientes com doença falciforme, independentemente do genótipo.

Apesar dos avanços no manejo da talassemia maior, a doença cardíaca continua sendo a principal causa de mortalidade em pacientes portadores desta doença.^15^

O envolvimento cardíaco na talassemia abrange um espectro de distúrbios que inclui disfunção miocárdica, arritmias, hipertensão e doença vascular periférica.^16^ Embora a siderose cardíaca (acúmulo de ferro nos miócitos cardíacos) como consequência de repetidas transfusões de sangue é considerada o principal fator etiológico para disfunção miocárdica em pacientes dependentes de transfusão, outros mecanismos fisiopatológicos são cada vez mais reconhecidos, especialmente nos pacientes não dependentes de transfusão.^17^ O manejo das complicações cardíacas na talassemia maior depende do tratamento da fisiopatologia subjacente, a qual muitas vezes é a sobrecarga de ferro.

A suscetibilidade à toxicidade ao ferro e suas manifestações fenotípicas variam muito entre os pacientes com talassemia. Atualmente, a detecção de deposição de ferro miocárdico por meio da ressonância magnética cardíaca continua sendo o melhor marcador de disfunção cardíaca futura.^18^

Estudos ecocardiográficos sugerem que a deposição miocárdica pode afetar diretamente a contratilidade ventricular esquerda, enquanto em outros casos pode causar restrição miocárdica do ventrículo esquerdo com hipertensão pulmonar concomitante e insuficiência cardíaca predominante à direita.^19^

As formas mais raras de Anemias Hemolíticas têm que ser pesquisadas pois apresentam características particulares que envolvem seus acompanhamentos clínicos.^20^

## References

[1] Brasil. Ministério da Saúde (2007). Departamento de Atenção Especializada.

[2] Brasil (2001). ANVISA.

[3] Brasil. Ministério da Saúde (2016). Departamento de Atenção Especializada e Temática.

[4] Brasil. Ministério da Saúde Secretaria de Atenção à Saúde Departamento de Atenção Hospitalar e de Urgência. Coordenação-Geral de Sangue e Hemoderivados. Área de Assessoramento Técnico às Talassemias.

[5] Weatherall DJ (2010). The inherited diseases of hemoglobin are an emerging global health burden. Blood.

[6] Chaturvedi S, DeBaun MR (2016). Evolution of sickle cell disease from a life-threatening disease of children to a chronic disease of adults: The last 40 years. Am J Hematol.

[7] Fitzhugh CD, Lauder N, Jonassaint JC, Telen MJ, Zhao X, Wright EC (2010). Cardiopulmonary complications leading to premature deaths in adult patients with sickle cell disease. Am J Hematol.

[8] Gladwin MT, Sachdev V, Jison ML, Shizukuda Y, Plehn JF, Minter K (2004). Pulmonary hypertension as a risk factor for death in patients with sickle cell disease. N Engl J Med.

[9] Sachdev V, Machado RF, Shizukuda Y, Rao YN, Sidenko S, Ernst I (2007). Diastolic dysfunction is an independent risk factor for death in patients with sickle cell disease. J Am Coll Cardiol.

[10] Cabrita IZ, Mohammed A, Layton M, Ghorashian S, Gilmore A, Cho G (2013). The association between tricuspid regurgitation velocity and 5-year survival in a North West London population of patients with sickle cell disease in the United Kingdom. Br J Haematol.

[11] Nagel RL, Fabry ME, Steinberg MH (2003). The paradox of hemoglobin SC disease. Blood Rev.

[12] Lionnet F, Hammoudi N, Stojanovic KS, Avellino V, Grateau G, Girot R (2012). Hemoglobin sickle cell disease complications: a clinical study of 179 cases. Haematologica.

[13] Chawla A, Sprinz PG, Welch J, Heeney M, Usmani N, Pashankar F (2013). Weight status of children with sickle cell disease. Pediatrics.

[14] Kim SH, Després JP, Koh KK (2016). Obesity and cardiovascular disease: friend or foe?. Eur Heart J.

[15] Fattizzo B, Giannotta JA, Cecchi N, Barcellini W (2021). Confounding factors in the diagnosis and clinical course of rare congenital hemolytic anemias. Orphanet J Rare Dis.

[16] Rund D, Rachmilewitz E (2005). Beta-thalassemia. N Engl J Med.

[17] Hershko C, Link G, Cabantchik I (1998). Pathophysiology of iron overload. Ann N Y Acad Sci.

[18] Wood JC (2008). Cardiac iron across different transfusion-dependent diseases. Blood Rev.

[19] Cappellini MD, Cohen A, Porter J, Taher A, Viprakasit V (2014). Guidelines for the Management of Transfusion Dependent Thalassaemia (TDT) [Internet].

[20] Kremastinos DT (2001). Heart failure in beta-thalassemia. Congest Heart Fail.

[21] Lopes A, Dantas MT, Ladeia AMT (2022). Prevalence of Cardiovascular Complications in Individuals with Sickle Cell Anemia and Other Hemoglobinopathies: A Systematic Review. Arq Bras Cardiol.

