# Supplementary material for: Hierarquizar a Anemia Falciforme nas Repercussões Cardíacas Sempre em Alerta ao Espectro Geral das Anemias Hemolíticas
Source: Arq Bras Cardiol. 2022 Nov 23;119(6):900–1. [Article in Portuguese] doi: 10.36660/abc.20220831 (PMC9814810; doi:10.36660/abc.20220831)
Supplement: Translation

# Translation: Sickle Cell Anemia as the First in the Hierarchy for Cardiac Alterations, Drives Attention to the Whole Spectrum of Hemolytic Anemias

**Affiliations:** 1 São Paulo SP Brazil Instituto do Coração da Faculdade de Medicina da Universidade de São Paulo - InCor-FMUSP, São Paulo, SP – Brazil; 2 São Paulo SP Brazil Departamento de Nefrologia - Beneficência Portuguesa de São Paulo, São Paulo, SP – Brazil

**Keywords:** Cardiovascular Diseases, Anemia, Sickle Cell, Anemia, Hemolytic, Hemonoglobinopathies/complications: Hypertension, Pulmonary/complications, Diagnostic, Imaging/methods

The focus on the cardiac repercussions of Hemolytic Anemias has been greatly emphasized by publications issued by academic and official government documents that are easily accessed by all health professionals, mainly articles that aim at Sickle Cell Anemia^1,2^ and Thalassemia.^3,4^

Sickle cell disease is the most frequent genetic hemoglobinopathy worldwide.^5^ Thanks to the improvement of the medical management of these patients, their life expectancy has improved in recent years.^6^ However, cardiopulmonary complications remain one of the main causes of death in adult patients with sickle cell disease.^7^

Current knowledge about cardiac involvement in sickle cell disease is mainly derived from studies of sickle cell anemia genotypes.^8,9^ Pulmonary systolic hypertension, assessed by increased tricuspid regurgitation rate (TRV) and left ventricular diastolic dysfunction diagnosed by ultrasound, has been associated with increased mortality and is characteristic of sickle cell anemia characterized by hemoglobin S^8-10^ homozygosis. On the other hand, hemoglobin SC disease (HbSC), resulting from compound heterozygosity for two different mutations of the beta-globin gene, has a different pathophysiology^11^ and a more attenuated clinical profile of clinical presentation.^12^ Patients with HbSC usually have a relatively low hemolysis rate and only mild anemia.^11^ In addition, the prevalence of obesity is higher in patients with HbSC than in sickle cell anemia.^13^ This non-hematological comorbidity may contribute to cardiac remodeling.^14^ Currently, echocardiography is recommended for routine follow-up of all patients with sickle cell disease, regardless of genotype.

Despite advances in the management of thalassemia major, heart disease remains the main cause of mortality in patients with this disease.^15^

Cardiac involvement in thalassemia encompasses a spectrum of disorders, including myocardial dysfunction, arrhythmias, hypertension and peripheral vascular disease.^16^ Although cardiac siderosis (accumulation of iron in cardiac myocytes) as a consequence of repeated blood transfusions is considered the main etiological factor for myocardial dysfunction in transfusion-dependent patients, other pathophysiological mechanisms are increasingly recognized, especially in patients not dependent on transfusion.^17^ Managing cardiac complications in thalassemia major depends on treating the underlying pathophysiology, which is often iron overload.

Susceptibility to iron toxicity and its phenotypic manifestations vary widely among thalassemia patients. At present, the detection of myocardial iron deposition by cardiac magnetic resonance imaging remains the best marker of future cardiac dysfunction.^18^

Echocardiographic studies suggest that myocardial deposition can directly affect left ventricular contractility, while in others, it may cause myocardial restriction of the left ventricle with concomitant pulmonary hypertension and predominant right heart failure.^19^

The rarest forms of Hemolytic Anemias have also to be investigated because they present particular characteristics that involve their clinical follow-ups.^20^
